# Difference in effects of open and closed motor skills on energy expenditure and after-burn effect in college student within physical classes

**DOI:** 10.7717/peerj.20562

**Published:** 2026-02-06

**Authors:** Xue Sun, Kuo Li, Yan Cao, Tao Zhang, Fang Yuan

**Affiliations:** 1 Physical Education Department, Beijing University of Posts and Telecommunications, Beijing, China; 2 Heze Hospital, Shandong Provincial Hospital, Shandong, China; 3 School of Humanities, Beijing University of Posts and Telecommunications, Beijing, China

**Keywords:** Physical activity, College student, Fat reduction, Energy expenditure, Metabolic substrates

## Abstract

**Introduction:**

Metabolic health is closely related to physical activity, and different types of motor skills may induce distinct physiological responses. To investigate the differences in energy expenditure and substrate metabolic characteristics between university students engaged in closed motor skills and open motor skills physical education classes, and to provide exercise prescription and theoretical reference for metabolic health promotion.

**Methods:**

Thirty-six male university students (*n* = 36, year = 20.83 ± 1.98) were recruited and asked to perform sequential open motor skills exercise (cricket practice, time = 40 min) and closed motor skills exercise (closed motor skills, time = 40 min) during university physical education classes. Resting energy expenditure (REE), exercise energy expenditure (EEE), energy expenditure rate (EER), respiratory quotient (RQ), fat energy supply rate (FESR), fat energy supply proportion (FESP), fat oxidation amount (FOA), fat oxidation rate (FOR), sugar energy supply rate (SESR), sugar energy supply proportion (SESP), sugar oxidation amount (SOA), sugar oxidation rate (SOR), rating of perceived exertion (RPE) and feeling scale (FS) was measured before exercise, during exercise in class, during a 3-h recovery period after class, and for 4 consecutive days after class.

**Results:**

EEE, FOA, and FESP were significantly lower (*P* < 0.01) for open motor skills but higher (*P* < 0.01) for SESP than for closed motor skills during in-class practice. Within 3 h of recovery after class, EEE, FOA, FESP, and FS were significantly higher for open motor skills than for closed motor skills (*P* < 0.05), but lower for SOA, SOA, SESP, and RPE than for closed motor skills (*P* < 0.01). Within 4 consecutive days after training, the REE for open motor skills was higher than pre-training (*P* < 0.05) on days 1 and 2, and higher than closed motor skills (*P* < 0.05) on day 1. The RQ of open motor skills was lower than that of pre-exercise and closed motor skills on day 1 and day 2 after exercise (*P* < 0.01, *P* < 0.05), whereas both REE and RQ of closed motor skills were not significantly different from pre-exercise at 4 days.

**Conclusion:**

(1) Open and closed motor skills have similar effects in promoting lipid and glucose metabolism, but open motor skills have a higher perception of exercise experience. (2) During exercise, open motor skills are more dependent on glucose supply than closed motor skills. However, during the recovery period, open motor skills are more fat-fueled and glycogen is more likely to be resynthesized to replenish depleted glycogen during exercise. (3) Open motor skills are superior to closed motor skills in increasing resting metabolic levels and fat metabolism efficiency.

## Introduction

Metabolic health plays a critical role in the maintenance of overall health and fitness, with the level of energy metabolism and the secretion of metabolic substrates as an important measure (Liu et al., 2024). Metabolic health affects not only our performance during exercise, but also our daily lives. Through effective metabolic pathways, the body converts the nutrients we consume into the energy needed for exercise and thought, thereby maintaining basic physiological functions (Dos Santos et al., 2024). On the other hand, metabolic substrates, such as glucose, fatty acids, and amino acids, are important factors that reflect the health status of an individual’s body. They not only provide fuel for the body, but also play an important role in regulating processes such as blood glucose, lipid metabolism, and muscle synthesis (Hengist et al., 2024). However, an abnormal metabolic profile can lead to the development of symptoms such as obesity, abnormal body image, and metabolic syndrome (Siroux et al., 2024). According to the World Health Organization (WHO), more than one billion people worldwide are obese. This includes 650 million adults, 340 million adolescents, and 39 million children. The data also shows that 43% of adults were overweight in 2022 ((NCD-RisC) NRFC, 2024). Therefore, how to effectively improve the metabolic health of individuals has become one of the hot topics in the field of medicine and exercise.

American College of Sports Medicine (ACSM) in *ACSM’s Guidelines for Exercise Testing and Prescription* shows that rational exercise can effectively promote the energy metabolism level and metabolic substrate secretion status of individuals (Thompson et al., 2013), and can also induce Excess Post-Exercise Oxygen Consumption (EPOC), the so-called after-burn effect, which refers to the elevated oxygen uptake and caloric expenditure that persists after exercise as the body restores homeostasis. This physiological mechanism contributes to preventing obesity and enhancing fat loss and body shaping (McCarthy et al., 2024). However, recent studies confirm that different types of exercise induce distinct physiological responses. Based on the classic classification by Poulton (1957), exercises can be divided into open and closed motor skills—a framework still widely used today (Voelcker-Rehage, 2008). Motor skills are commonly classified into open and closed types based on the predictability of the environment in which they are performed. Closed motor skills occur in stable and predictable settings, allowing athletes to execute movements with minimal external interference, such as in running or swimming (Qiu, Zhai & Chen, 2024). In contrast, open motor skills are performed in dynamic and changing environments, requiring individuals to continuously adapt their actions in response to external stimuli, such as in football or basketball (Formenti et al., 2021; Gu et al., 2019). Currently, most studies have compared the metabolic effects of closed motor skills programs with varying intensities, such as high-intensity interval training (HIIT), which alternates short bursts of intense exercise with recovery periods, and sprint interval training (SIIT), which involves repeated all-out sprints with longer rest intervals (González-Gálvez et al., 2024). Some have also examined resistance training and Tai Chi (Chang & Liu, 2024), but few studies have directly compared different types of exercise programs. For example, Maillard et al. (2016) compared HIIT and moderate-intensity continuous training (MICT) effects on metabolic health, yet the number of trials comparing different motor skill types remain limited.

In summary, to investigate the differences in energy metabolism, metabolic substrates, and afterburn effect on individuals with different types of exercise programs and to evaluate the efficiency of exercise, the present study, based on the studies of Jiang et al. (2024) and Wang et al. (2024), for the first time included open motor skills and closed motor skills as independent variables in the experiment and evaluated the differences in energy expenditure, metabolic substrate effects, and induced EPOC of the two skills on individuals within the same time period (one college physical education class). The effects of the two exercises on the students’ subjective perceptions, including exercise experience and fatigue, were also analyzed. The goal is to provide optimal exercise prescription and theoretical references for promoting metabolic health, preventing metabolic syndrome, and cultivating exercise habits in college students.

## Materials and Methods

### Participants

Thirty-six healthy male undergraduates (aged 18–23 years) from Beijing University of Posts and Telecommunications participated in this study. Sample size was calculated using G*Power software, with an assumed medium effect size (f = 0.27), α = 0.05, and power (1 − β) = 0.81 for repeated-measures analysis of variance (ANOVA). Inclusion criteria were non-athletes with no history of smoking, diabetes, or metabolic disorders. Participants were screened *via* standard medical examination to exclude any contraindications for high-intensity exercise. Eligible participants were then randomly assigned to intervention sequences using a computer-generated randomization schedule. All subjects gave written informed consent after being fully informed of the study aims and procedures. Demographic details are summarized in Table 1.

**Table 1:** Participants’ physical characteristics.

Parameter	Value
Number (*n*)	36
Height (cm)	177.2 ± 4.8
Weight (kg)	70.2 ± 6.1
BMI (kg/m^2^)	22.4 ± 1.5
Body fat ratio (%)	14.8 ± 3.2
VO_2_max (ml/min/kg)	43.6 ± 4.3

### Procedures

All participants took part in a randomized crossover-controlled trial. One week before the sessions, we assessed height, weight, body composition (using bioelectrical impedance), VO_2_max, and peak power output. Body composition, weight, and body mass index (BMI) were measured at the Physical Fitness Measurement Center of Beijing University of Posts and Telecommunications. VO_2_max and peak power output were tested in the exercise physiology laboratory.

VO_2_max test was performed on a calibrated electronically braked cycle ergometer (Lode Excalibur Sport) with breath-by-breath gas analysis (Cosmed Quark CPET). Calibration was done before each test using standard gas mixtures and a 3-L syringe. Participants avoided caffeine, alcohol, and vigorous exercise for 24 h and fasted for at least 3 h. After a 5-min warm-up at 50 W, we applied a ramp protocol increasing by 25 W per minute until volitional exhaustion (Glaab & Taube, 2022). VO_2_max attainment was confirmed if at least two of the following criteria were met: a plateau in VO_2_ despite increased workload, RER ≥ 1.10, or heart rate reaching ≥90% of their age-predicted maximum (220-age). The 5-min cool-down at 30 W completed the test (Sperlich et al., 2015).

Written informed consent was obtained from all participants prior to the commencement of the study. The study procedures were explained in detail, including potential risks and benefits, and all participants signed consent forms voluntarily. All procedures adhered to the NIH guidelines and were approved by the Human Research Ethics Committee of Beijing University of Posts and Telecommunications (Approval No. TY0839202309).

#### Baseline testing

A ramp protocol on a cycle ergometer established each participant’s VO_2_max. Power output began at 70 W and increased by 10 W each minute, with cadence fixed at 60 RPM, progressing through up to twenty stages or until the rider reached exhaustion. This study referenced Nicole’s classic exercise intensity baseline modeling method. During exercise, HR was recorded using the POLAR Verity Sense portable HR monitoring armband, with a sampling frequency of 1 time/s (Olney et al., 2018). Finally, workloads corresponded to 25%, 50%, and 90% of each individual’s HRmax were prescribed for both the cricket (open skills) and continuous (closed motor skills) exercise sessions.

#### Exercise protocol

To ensure objectivity and minimize potential bias, a single-blind design was employed in this study, where participants were unaware of the specific exercise intensity targets to prevent conscious alteration of effort. HR during the intermittent cricket drill was continuously monitored and maintained within the 60–85% HRmax range using POLAR heart rate armbands, allowing real-time adjustments as needed. The exercise protocol was implemented in the form of a public physical education class for university students, which included the closed motor skills of cricket practice, respectively, both lasting 40 min.

The cricket training program is based on Nutt et al.’s (2022) training program, which includes cricket-specific sprint performance (running between the wickets, two runs), cricket-specific running preparation (6 × 35.36 m sprints including a 180° change of direction), and LIFT jump, also known as the burpee, is a full-body exercise involving a squat, plank, and jump in one continuous movement. It enhances cardiovascular fitness, strength, and coordination, making it common in high-intensity training (6 × 4 repetitions up to 85% of a repetition maximum). Rest periods during each set were limited to no more than 5 min, while exercise intensity was controlled between 60–85% HRmax.

The closed-loop skills refer to the protocol of Patten et al. (2022), in the form of a jogging exercise in the school stadium (international standard athletic field), lasting a total of 30 min without any intermittent activity during the process, with the training intensity controlled between 50–70% HRmax. The rest of the time was spent in a 5-min warm-up and 5-min autonomous cool-down.

#### Methodological controls

Before the experiment, participants’ dietary structure and food intake from the previous week were recorded using a standardized 7-day dietary recall questionnaire, and this dietary pattern was replicated post-intervention to minimize variability. The test was conducted over four days, with each session scheduled between 3:00 p.m. and 6:00 p.m. Participants were familiarized with all procedures and equipment prior to baseline measurements. On each test day, subjects arrived at the laboratory in the morning and, after standardized hydration, completed a 30-min REE assessment in the supine position. In the afternoon, they performed the assigned exercise protocol while heart rate and metabolic data were continuously monitored. Gas exchange was analyzed immediately post-exercise, at 3 h post-exercise, and on each of the following four mornings at the same time as the initial REE test. During the 3-h recovery period, participants consumed 200 mL of purified water during the first 5 min of each hour and refrained from additional physical activity. Prior to each testing session, the gas analyzer’s sensors—including temperature, humidity, pressure, flow rate, and O_2_ and CO_2_ concentrations—were carefully calibrated, and procedures began only after confirming stable baseline readings.

### Measurements

#### Body morphology measurement

The subjects’ basic physiological and morphological parameters, including height, weight, BMI, and body fat ratio, were measured by an automated height and body mass meter (HGM-1700) with an accuracy of 0.1 cm and 0.1 kg. The body fat ratio was measured using a human body composition analyzer (Inbody 720; Biospace, Seoul, South Korea), which requires that subjects do not engage in strenuous physical activity for 8 h prior to the test and not eat or drink for 2 h prior to the test.

#### Level of energy metabolism at resting

Resting metabolic indices included BMR and resting RQ. Subjects arrived at the laboratory before 6:30 a.m. on an empty stomach and walked slowly (≤200 m). Upon arrival, they remained seated quietly for more than 30 min to ensure physiological stabilization. Afterward, they lay in a supine position, and REE was measured for 30 min using a gas analyzer.

The device judged the body to have entered the resting state based on VO2 fluctuating up and down by no more than 5% for 5 consecutive minutes. Test Environment: Room temperature was maintained at 22 °C, relative humidity at 50%. The oxygen consumption (VO2) and carbon dioxide output (VCO2) of the subjects were selected for 30 min, and the RQ values were calculated for 30 min.

RQ was calculated according to the following formula (Feng, 2006):

$RQ = VCO2 \div VO2.$

#### Post-exercise metabolic status and after-burn effect

The Gas Metabolizer was worn continuously for 10 min before, during, and during the recovery period, and respiratory gas parameters were collected in real time. In this study, based on José’s research, the recovery period was defined as resting for more than 30 min after exercise (Vilaça-Alves et al., 2016). At the end of the test, the data collected was processed using the Gas Metabolizer software. The subjects VO2 and VCO2 were selected for 1 min and summed to obtain the total amount of VO2 and VCO2 in a given time period. Resting energy expenditure (REE), exercise energy expenditure (EEE), energy expenditure rate (EER), respiratory quotient (RQ), fat energy supply rate (FQ), and fat energy supply rate (FQ) were calculated based on the VO2 and VCO2 totals. fat energy supply rate (FESR), fat energy supply percentage (FESP), fat energy supply amount (FESA), fat oxidation amount (FOA), fat oxidation rate (FOR), sugar energy supply amount (SESA), sugar energy supply rate (SESR), sugar energy supply percentage (SESP), sugar oxidation amount (SOA), sugar oxidation rate (SOR). oxidation rate (SOR).

The formula is as follows (Frayn, 1983; Trombold et al., 2013):

$EER\; \left( {kcal/min} \right) = 3.716 \times \left( {VO2\; L/min} \right)\; + 1.332 \times \left( {VCO2\; L/min} \right)$

$EEE\; \left( {kcal} \right) = \left[ {3.716 \times \left( {VO2\;L/min} \right) + 1.332 \times \left( {VCO2\;L/min} \right)} \right] \times time\; \left( {min} \right)$

$SESR\; \left( {kcal/min} \right) = \left( {\left( {\left( {NPRQ-0.707} \right) \div 0.293} \right) \times 100} \right) \times EER$

$SOR\; \left( {g/min} \right) = 4.585\; \times \left( {VCO2\; L/min} \right){\rm -}3.2255 \times \left( {VO2\; L/min} \right)$

$SOA\; \left( g \right) = SOR\; \left( {g/min} \right) \times time\; \left( {min} \right)$

$FESR\; \left( {kcal/min} \right) = EER-SOR$

$FOR\; \left( {g/min} \right) = 1.6946 \times \left( {VO2\;L/min} \right){\rm -}\left( {VCO2\; L/min} \right)$

$FOA\; \left( g \right) = FOR\; \left( {g/min} \right) \times time\left( {min} \right)$

Note: exercise energy expenditure (EEE), energy expenditure rate (EER), fat energy supply rate (FESR), fat oxidation amount (FOA), fat oxidation rate (FOR), sugar energy supply rate (SESR), sugar oxidation amount (SOA), sugar oxidation rate (SOR).

#### Perceptual response evaluation

Participants’ overall exercise impressions were measured with Hardy and Rejeski’s Feeling Scale (FS) alongside the 10-point Borg Rating of Perceived Exertion (RPE) (Borg, 1998; Hardy & Rejeski, 1989). Before the experiment begins, participants are instructed on how to use the FS and RPE. Among these, FS consists of 10 levels ranging from −5 to 5, representing participants’ immediate mood and exercise experience during the activity. This scale uses a positive scoring method, with 0 as the baseline; higher scores indicate a better experience. RPE is used to assess participants’ current fatigue levels. This scale also has 10 levels ranging from 0 to 10, with 0 as the baseline; higher scores indicate greater fatigue. In the 15 min following each workout, participants gave verbal FS and RPE ratings every minute, which the researcher recorded to evaluate their perceived exertion and affective response (Ekkekakis & Petruzzello, 2002).

### Statistical analysis

Data was analyzed in SPSS 17.0 and are presented as mean ± SD. Each variable’s distribution was checked with the Shapiro-Wilk test. For normally distributed measures (*e.g*., EEE, FOA, FOR, FESP, SOA, SOR, SESP, RPE, FS), a one-way repeated-measures ANOVA compared open motor skills *vs* closed motor skills responses at identical time points and throughout the 3-h recovery. REE and respiratory quotient (RQ) before exercise and on day 4 post-exercise were examined *via* two-way repeated-measures ANOVA to assess group, time, and group × time interaction effects; significant interactions prompted Tukey’s pairwise comparisons between groups at each stage and between pre- and post-exercise values. When data violated normality, the Friedman test served as the nonparametric alternative. Statistical significance was set at *P* < 0.05.

## Results

No adverse events occurred. One participant withdrew during the closed motor skills session, giving 97% adherence, while all subjects completed the open motor skills session, resulting in 100% adherence.

### Characterization of energy expenditure in closed *vs* open motor skills during post-exercise and recovery periods

Work performed during open skills exercise was significantly lower than closed motor skills exercise (F = 68.15, *P* < 0.01, 95% CI [−22.01 to −14.67]), with power during open skills exercise being 178.94 ± 15.12 W (90% VO_2_max) and 56.37 ± 5.54 W (25% VO_2_max), respectively; actual power during closed motor skills exercise was 103.87 ± 9.5 W. Energy expenditure during exercise was significantly lower for open motor skills than for closed motor skills (F = 98.45, *P* < 0.01, 95% CI [−14.25 to −9.67]), but significantly higher for open motor skills than for closed motor skills during the 3-h recovery period (F = 28.43, *P* < 0.01, 95% CI [4.31–7.49]). VO2 and VCO2 were significantly lower in open skills exercise than in closed motor skills (F = 52.47, *P* < 0.01, 95% CI [−6.38 to −2.23]), VO2 was significantly higher in open skills recovery than in closed motor skills (F = 18.45.47, *P* = 0.034, 95% CI [1.66–3.18]), and VCO2 was not significantly different in both groups. As shown in Table 2.

**Table 2:** Metabolic differences in closed *vs* open skills exercise.

		Open-skill exercise	Closed-skill exercise	*P*-value
Work in exercise (kJ)		242.64 ± 35.49**	410.4 ± 55.1	<0.01
Power in exercise (W)	Peak	178.94 ± 15.12 W	103.87 ± 9.5	<0.01
	Mini	56.37 ± 5.54		
Energy consumption in exercise (kcal)		474.34 ± 64.05**	631.84 ± 72.37	<0.01
Energy consumption during recovery (kcal)		434.28 ± 42.36**	351.48 ± 37.49	<0.01
VO2 during exercise (L)		72.36 ± 14.78**	121.36 ± 22.34	<0.01
VCO2 during exercise (L)		81.56 ± 14.68**	108.91 ± 22.13	<0.01
VO2 during recovery (L)		75.18 ± 12.52*	55.94 ± 12.41	0.034
VCO2 during recovery (L)		54.69 ± 11.26	48.78 ± 7.32	0.628

**Note:**

* *P* < 0.05.

** *P* < 0.01.

VO2, Oxygen consumption; VCO2, carbon dioxide output.

### Differential expression of metabolic substrates in closed *vs* open skills movement

**Figure 1:**
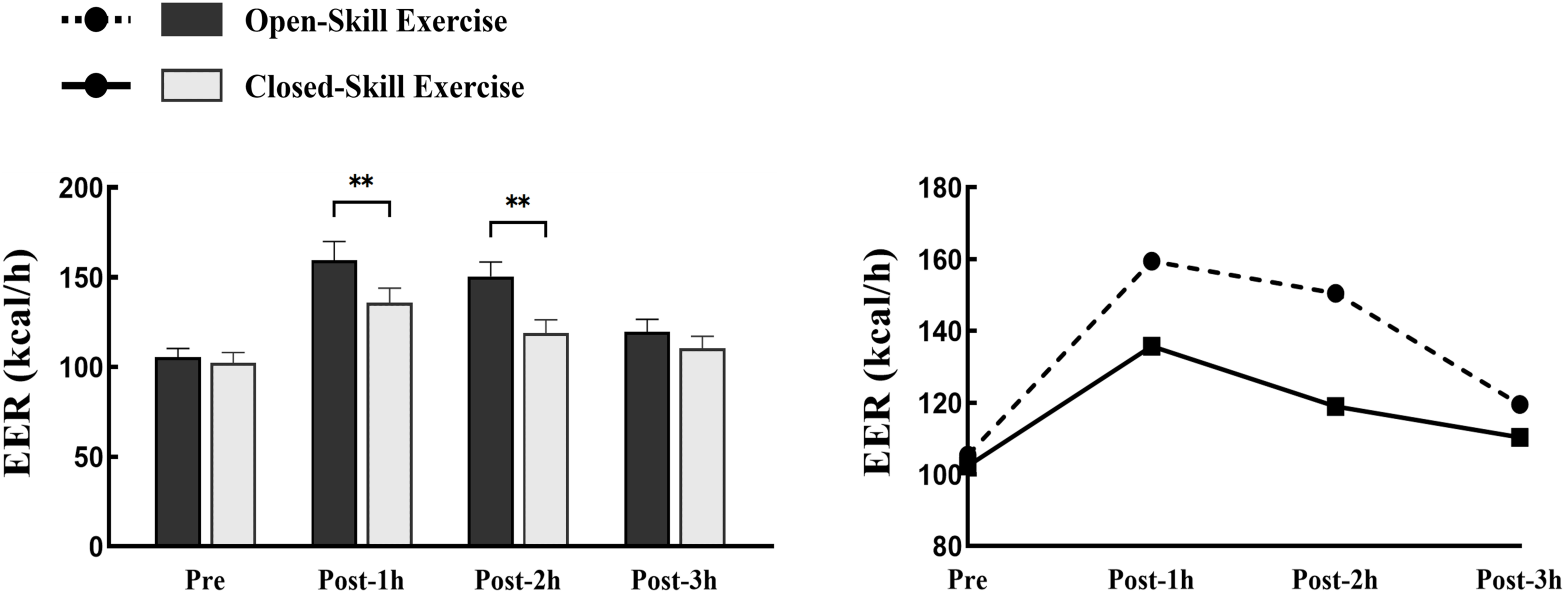
Time-domain characterization of energy expenditure rates in two pre-exercise and recovery periods. **P < 0.01; RPE, rating of perceived exertion; FS, feeling scale.

The EER was significantly higher for open motor skills than for closed motor skills during the first *vs* the second hour of recovery (F = 44.52, *P* < 0.01, 95% CI [3.68–8.54]); pre- and post-exercise comparisons showed that the EER was significantly higher for open motor skills during the recovery period than before the exercise (F = 61. 22, *P* < 0.01, 95% CI [1.02–3.24]; F = 14.35, *P* = 0.027, 95% CI [0.58–2.03]); whereas closed motor skills had significantly higher EER than pre-exercise only in the first hour of the recovery period (F = 20.18, *P* = 0.039, 95% CI [0.26–1.09]), and EERs in the second and third hours were not significantly different from pre-exercise (see Fig. 1).

**Figure 2:**
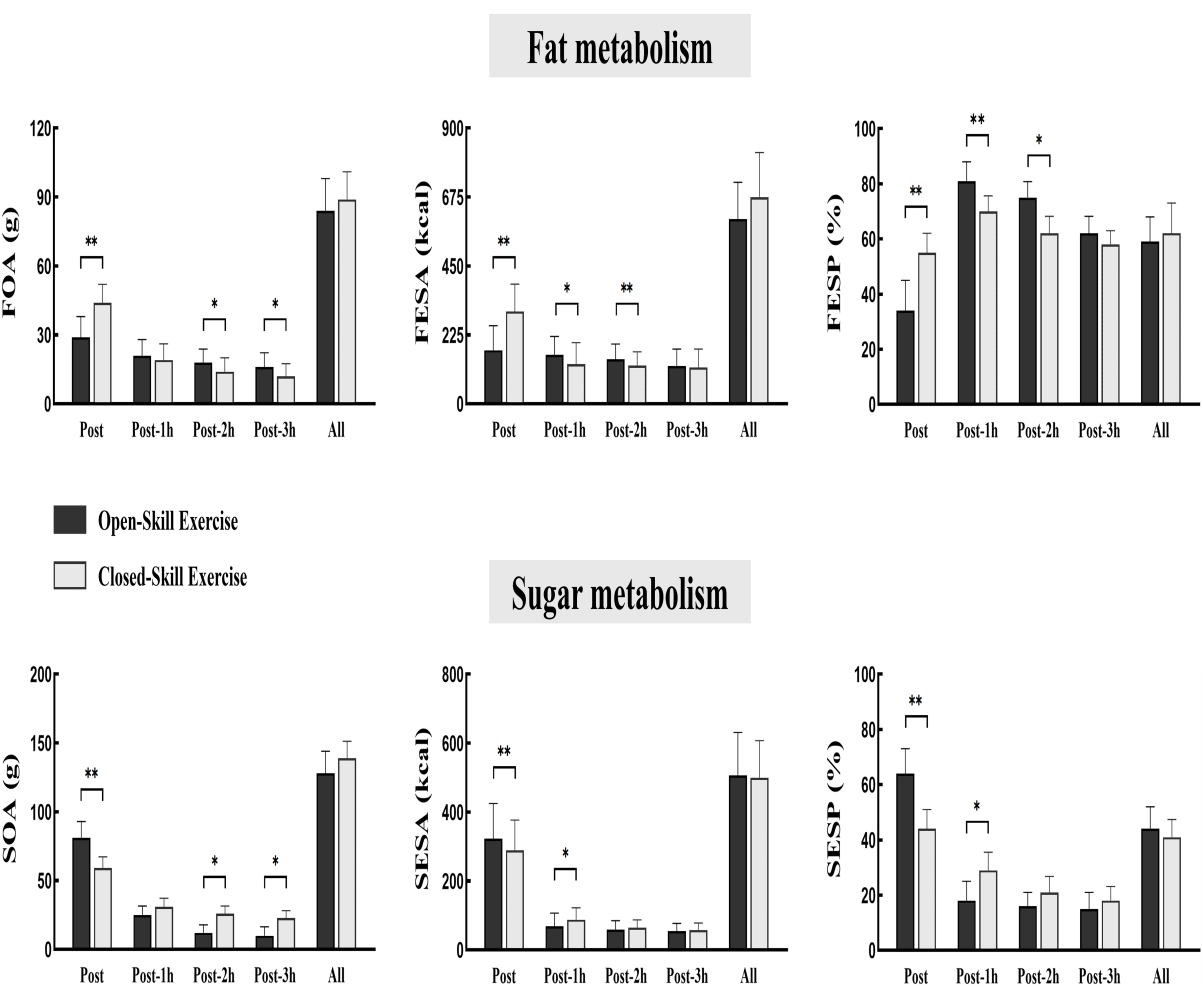
Fat oxidation and glucose oxidation levels during two types of exercise and recovery. *P < 0.05, **P < 0.01; RPE, rating of perceived exertion; FS, feeling scale.

The results of substrate metabolism during exercise and recovery showed that for lipid metabolism, FOA, FESA and FESP were significantly lower in open motor skills exercise than in closed motor skills exercise (F = 71.03, *P* < 0.01, 95% CI [−2.24 to −0.89]). However, during the recovery period, all the above indexes were significantly higher in open motor skills than in closed motor skills (F = 11.39, *P* = 0.021, 95% CI [0.53–1.41]; F = 63.23, *P* < 0.01, 95% CI [1.05–2.67]). Regarding glucose metabolism, SOA and SESA were not significantly different from closed-loop exercise, but SESP was significantly higher than closed-loop exercise (F = 33.74, *P* < 0.01, 95% CI [0.23–2.36]). After 1 h of recovery, SOA was significantly lower in the open skills group than in the closed motor skills group (F = 13.12, *P* = 0.041, 95% CI [−2.31 to −0.65]), and SESA and SESP were significantly lower than in the closed motor skills group (F = 39. 22, *P* < 0.01, 95% CI [−2.18 to −1.09]); at 2 and 3 h of recovery they were significantly lower than in the closed motor skills (F = 22.68, *P* < 0.01, 95% CI [−1.31 to −0.05]). There was no significant difference between closed and open motor skills for FOA, FESA, FESP, SOA, SESA, and SESP for “exercise + recovery period” (see Fig. 2).

### Effects of closed *vs* open skills exercise on resting metabolism and after-burn effect

**Figure 3:**
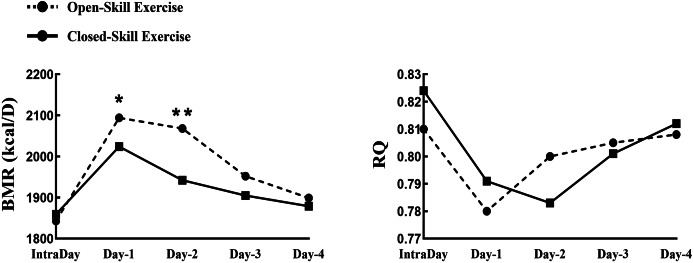
Differences in resting metabolic level before and four consecutive days after two exercise conditions. *P < 0.05, **P < 0.01; RPE, rating of perceived exertion; FS, feeling scale.

The results of resting metabolic values after exercise showed that REE was significantly higher than pre-exercise basal values on days 1 and 2 after open skills (F = 11.46, *P* = 0.041, 95% CI [0.18–1.07]); REE on day 1 after open skills was significantly higher than REE on day 1 after closed motor skills (F = 16. 71, *P* = 0.035, 95% CI [0.43–1.22]); On the first day after open skills training, the RQ value was significantly lower than that after closed motor skills training and before open skills training. On the first day after open skills training, the RQ value was significantly lower than that after closed motor skills training and before open skills training (F = 8.93, *P* = 0.047, 95% CI [0.09–0.51]); and RQ on day 2 after open skills was significantly lower than before closed motor skills and before open skills training (F = 12.66, *P* = 0.040, 95% CI [0.13–0.94]) (see Fig. 3).

### Differences in subjective feelings in closed *vs* open motor skills movement

**Figure 4:**
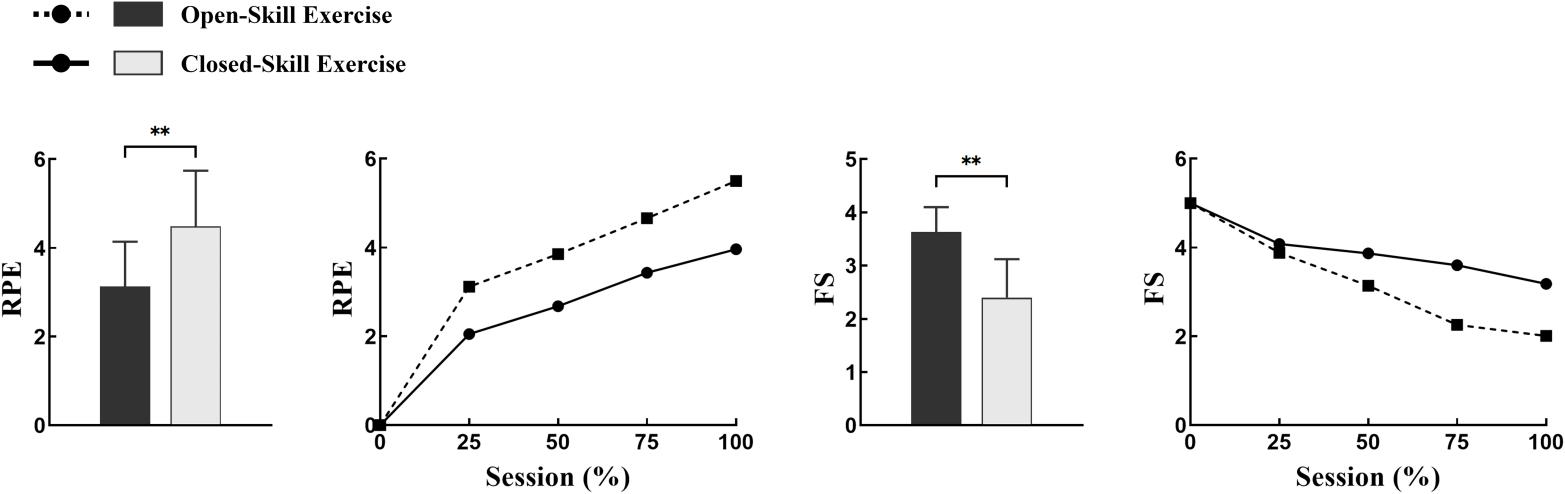
Differences in perceived responses in open *vs* closed motor skills campaigns. **P < 0.01; RPE, rating of perceived exertion; FS, feeling scale.

In terms of RPE, overall subjective fatigue was lower for open motor skills than for closed motor skills (F = 36.14, *P* < 0.01, 95% CI [0.84–1.55]). During the exercise, RPE in open motor skills was not equal across the four phases, showing no significant change at the beginning of the exercise (pre-25%), but a small increase in the middle and late phases (50–75%) (*P* = 0.042). In contrast, for closed motor skills, RPE showed a continuous increase (*P* < 0.05) from the 25% stage and peaked at the end of the exercise. Regarding FS, the overall experience was higher in open motor skills than in closed motor skills (F = 29.01, *P* < 0.01, 95% CI [0.88–1.69]). For open motor skills, FS showed only a small decrease at the 75% level (*P* = 0.038), with no significant change thereafter. Closed motor skills, on the other hand, showed a continuous decrease (*P* < 0.05), with the most significant decrease (*P* = 0.025) during the middle and late stages of the exercise (50–75%), reaching its lowest value at the end of the exercise. As shown in Table 3 and Fig. 4.

**Table 3:** Metabolic differences in closed *vs* open skills exercise.

	Session	Open-skill exercise	Closed-skill exercise
RPE	Mean	3.13 ± 1.01**	4.48 ± 1.26
	25%	2.05 ± 0.83	3.12 ± 0.87
	50%	2.68 ± 0.77	3.85 ± 0.59
	75%	3.43 ± 1.06	4.66 ± 0.68
	100%	3.96 ± 0.81	5.50 ± 1.12
FS	Mean	3.63 ± 0.47**	2.40 ± 0.72
	25%	4.08 ± 0.55	3.88 ± 0.63
	50%	3.87 ± 0.64	3.14 ± 0.49
	75%	3.60 ± 0.39	2.26 ± 0.87
	100%	3.18 ± 0.46	2.01 ± 0.65

**Notes:**

** *P* < 0.01.

RPE, Rating of perceived exertion; FS: feeling scale.

## Discussion

Closed motor skills and open motor skills sports are two different types of sports based on whether or not the external context changes during the movement. Some research has now confirmed that athletes in both types of sports have some differences in physical fitness and metabolic levels. However, these differences can be as small as a single training session. From an exercise science perspective, MICT is less intense, and subjects are more likely to adhere to it (Moncion et al., 2024). While cricket is generally considered to be HIIT, this type of exercise produces greater energy expenditure and a higher rate of fat oxidation, which has a positive effect on the prevention of metabolic diseases related to cardiovascular disease, obesity and diabetes, as well as a reduction in all-cause mortality (Forrest et al., 2020). And, crucially, the greatest benefit of open motor skills exercise is in EPOC, what we call the after-burn effect (Sturdy & Astorino, 2024). Therefore, in this article, we verified the difference in the effects of the two types of exercise on individual metabolism through repeated measures experiments.

This study found that open motor skills exercise elicited higher post-exercise metabolic responses and energy efficiency than closed motor skills exercise, despite lower energy expenditure during the activity. Notably, energy expenditure during the 3-h recovery was significantly higher in the open motor skills condition, indicating a stronger EPOC effect. This aligns with Jiang et al. (2024), who reported higher EPOC, FOR, and FESP after HIIT compared to MICT, especially in the early recovery phase, with FESP about 1.4 times greater. These findings support that open motor skills exercise induces greater total EEE and enhances fat metabolism during recovery. Although exercise duration was shorter, total energy expenditure (exercise + recovery) was similar between conditions, likely due to more sustained EPOC in open motor skills tasks. Energy expenditure remained elevated for three hours of post-exercise, whereas closed motor skills returned to baseline earlier. Wang et al. (2024) similarly found prolonged EPOC and FOR after Tabata-style open motor skills training. Thus, open motor skills exercise offers a time-efficient strategy to enhance metabolic activity and fat oxidation, making it especially suitable for sedentary or obese individuals seeking effective training adaptations.

The second major finding was that dominant metabolic substrates differed between open and closed motor skills exercise. During exercise, lipid metabolism indices (FOA, FESA, FESP) were significantly higher in closed motor skills exercise, indicating more efficient fat oxidation, while glucose contribution was significantly greater in open motor skills, suggesting a higher reliance on glycolytic pathways. However, during the 3-h recovery, open motor skills exercise showed significantly greater lipid metabolism than closed motor skills, as evidenced by increased FOA, FESA, and FESP. This shift suggests that the advantage of open motor skills exercise in promoting lipid oxidation primarily emerges post-exercise, whereas closed motor skills promote fat metabolism during activity. These results are supported by studies showing that open motor skills exercise can significantly lower postprandial triglycerides and very low-density lipoprotein (VLDL) levels, reflecting enhanced lipid clearance and metabolism (Maylor et al., 2018; Trombold et al., 2013). Further evidence from Kiens demonstrated that even after high-carbohydrate intake, fat oxidation remained elevated for up to 18 h post open motor skills exercise, likely due to the prioritization of glycogen resynthesis and consequent reliance on fat for energy during recovery (Kiens & Richter, 1998). Taken together, our findings demonstrate that open motor skills exercise elicits a delayed but prolonged enhancement in lipid metabolism, offering physiological advantages for recovery and metabolic regulation beyond the exercise bout itself—especially relevant for individuals aiming to improve fat utilization efficiency.

The third finding indicated that open skills exercise significantly elevated REE, particularly on the first day post-exercise, while closed motor skills exercise did not induce such changes across four consecutive days. This suggests that open skills exercise elicits a stronger and more sustained metabolic response. Physiologically, high-intensity or intermittent stimuli—as typically involved in open skills activities—can activate the autonomic nervous system and endocrine responses, thereby enhancing REE more effectively than moderate-intensity continuous activity (Boutcher, 2011; Gibala & McGee, 2008). This aligns with prior findings: Sevits et al. (2013) reported increased REE post-exercise, though inconsistent results across studies may stem from variations in subject profiles, exercise intensity, and total energy cost. Hunter et al. (1998) proposed that only intensities exceeding 70% VO_2_max, which induce substantial EPOC, can effectively elevate REE. Supporting this, Paoli et al. (2012) demonstrated that high-intensity intermittent resistance training maintained elevated REE 22 h post-exercise, surpassing moderate-intensity protocols. While Hazell et al. (2012) noted no difference in 24-h total oxygen consumption between modalities, they observed significantly higher post-exercise oxygen use following intermittent protocols. In our study, the elevated REE following open skills exercise may have lasted approximately 14–38 h—slightly below the 24–48-h elevation reported elsewhere—possibly due to subject characteristics (Hazell et al., 2012). Although REE on day 2 was not significantly higher, the subtle upward trend could still contribute to long-term energy balance, as even small daily surpluses in energy expenditure may help prevent metabolic disorders such as obesity (Bagheri et al., 2023).

The fourth finding of this study was that open motor skills elicited better exercise experiences and perceptual responses from individuals for the same amount of time. Previous evidence suggests that the subjective experience of exercise is important and has a direct impact on the development of adherence and interest in exercise (de Sampaio Barros et al., 2024). In the present study, although the exercise intensity of the open skills was slightly higher than that of the closed motor skills by about 10% HRmax for the same amount of time, it elicited higher FS with lower RPE in the subjects, which also means that due to the change in exercise form, the subjects did not show excessive fatigue and poorer sensation of exercise experience due to the difference in intensity. Combined with the above results on metabolism, we can find that open motor skills not only have higher EPOC and fat and weight loss efficiency, but also bring more compliance and interest to the exercisers.

The physiological mechanisms underlying these results may relate to the intensity and metabolic demands of open skills exercise. Although its duration was shorter, open skills induced greater glucose oxidative efficiency and a higher dependence on carbohydrate metabolism. Studies show that sugar oxidation rates in open skills sports like cricket are nearly three times higher than in aerobic activities of similar intensity (Romijn et al., 1993), and high-intensity bouts can rapidly deplete muscle glycogen—*e.g*., 1 min at 150% VO_2_max reduces glycogen by 20% (Ortega et al., 2015), and a single HIIT session can lower it to 28–37% of baseline (Gollnick et al., 1973). This depletion prompts the body to prioritize glycogen resynthesis post-exercise, supported by elevated insulin and glucose levels (MacDougall, Ward & Sutton, 1977), while sustaining high oxidative demand during activity (Little et al., 2011). The repeated anaerobic, aerobic transitions in open skills may further elevate the reliance on glycogen and trigger a compensatory increase in fat oxidation during recovery (Sakamoto, Ueda & Nakahara, 2024). This was evidenced by a significant and prolonged reduction in resting RQ values post-exercise, suggesting enhanced lipid metabolism efficiency (Little et al., 2011). Such changes may stem from improved mitochondrial function in skeletal muscle, promoted by open skills–induced gene expression, mitochondrial biogenesis, and signaling pathway activation (Pascoe & Gladden, 1996). Hormonal responses, such as elevated epinephrine, norepinephrine, and growth hormone (Herring et al., 2023; Nam et al., 2024), also inhibit lipogenesis and promote lipolysis and fat oxidation, contributing to the increased reliance on lipid substrates during post-exercise rest (Watanabe et al., 2022).

In conjunction with the results of this study, we compared substrate metabolic profiles during the recovery period after open and closed motor skills. Although the total FOA was 7.3% lower in open motor skills than in closed motor skills during the “exercise + recovery” period, there was no significant difference. Considering that open motor skills exercise is slightly more intense than closed motor skills exercise and thus corresponds to better resting fat oxidation, and that the EPOC of open motor skills exercise lasts at least 3 h, the actual difference in total fat oxidation between open motor skills and closed motor skills exercise should be less than 6.9%. The present study combines previous evidence to conclude that open and closed motor skills are similar in terms of exercise-induced overall fat metabolism efficiency during “exercise + recovery”, and that there is no significant difference between closed and open motor skills in terms of overall glucose metabolism during “exercise + recovery”. There was no significant difference between closed and open motor skills in terms of total glucose metabolism during “Exercise + Recovery” (Bellou et al., 2013). However, during exercise, open skills were more dependent on sugar for energy supply; during recovery, open skills used more fat than sugar for oxidative energy supply than closed motor skills. The changes in substrate metabolism characterized by open skills during exercise and recovery may be related to the fact that open skills induces changes in the number and activity of enzymes involved in lipid metabolism in skeletal muscle and induces increased efficiency of lipid metabolism (Seip & Semenkovich, 1998). In addition, open motor skills can lead to large glycogen depletion in a short period of time, and the post-exercise glycogen compensation mechanism may also have induced the characteristic tendency of glycogen resynthesis and lipid metabolism to catabolize and oxidize and provide energy after open motor skills (Trapp et al., 2008).

Therefore, based on the principle of motivation in sports (Schmid et al., 2020), this study argues that open skills sports, represented by cricket, should be used as a directed elective within the physical education classes of university students, and that such specialized exercise content should be used as the main teaching and practice activities within the classes (about 70% of the total class time). At the same time, according to the requirements of the Principle of Appropriate Load (Impellizzeri et al., 2022), MICT is organized as a part of basic physical training as a supplementary content in each class. This practice is more conducive to the development of exercise interest and exercise habits, while improving the metabolic level of college students and preventing obesity or metabolic syndrome.

## Limitations and suggestions for future research

Several constraints warrant consideration. Although both sexes were initially included, female adherence was below 30%, mainly due to hormonal fluctuations and menstrual cycle effects on exercise tolerance. For future studies, we recommend phase-based designs aligned with menstrual cycles, use of tracking apps for scheduling, and gender-specific protocols to address these factors and improve adherence. Second, the acute, cross-sectional design permits only immediate post-exercise comparisons; long-term adaptations remain unexamined. Full-time undergraduates exhibited low baseline activity and scheduling conflicts, resulting in poor compliance for any extended protocol and precluding a longitudinal framework.

This study collected dietary structure and food intake data using a standardized one-week dietary recall questionnaire. However, it is acknowledged that the one-week recall method may lack precision and is subject to recall bias, which could affect the accuracy of the dietary data. Future studies should consider employing more objective and comprehensive dietary assessment tools, such as multiple-day food diaries or direct observation, to minimize these limitations and improve data reliability.

To address these gaps, future studies will reintroduce female cohorts with improved adherence strategies. Rather than relying solely on tightened physical-status screening, we will adopt phase-based intervention timelines, menstrual cycle tracking, and gender-specific protocols to better accommodate physiological variability and enhance inclusivity. Moreover, repeated-measures protocols under controlled conditions will refine individual monitoring across exercise types. Finally, efforts will pivot toward a longitudinal intervention, leveraging tailored recovery schedules and motivational strategies to bolster adherence. This multifaceted approach aims to yield robust data on optimal exercise dosage for metabolic improvement and sustained exercise interest among university students.

## Conclusion

(1) Open and closed motor skills have similar effects in promoting lipid and glucose metabolism, but open motor skills have a higher perception of exercise experience. (2) During exercise, open motor skills are more dependent on glucose supply than closed motor skills. However, during the recovery period, open motor skills are more fat-fueled and glycogen is more likely to be resynthesized to replenish depleted glycogen during exercise. (3) Open motor skills are superior to closed motor skills in increasing resting metabolic levels and fat metabolism efficiency.

## Supplemental Information

10.7717/peerj.20562/supp-1 Supplemental Information 1 Raw Data.

## Additional Information and Declarations

### Data Availability

The following information was supplied regarding data availability:

The raw data is available in the Supplemental Files.
